# Cardiopulmonary resuscitation for lay people: Evaluation of videos from the perspective of digital health literacy

**DOI:** 10.1590/1518-8345.5623.3542

**Published:** 2022-07-15

**Authors:** Sara Rodrigues Vilela, Jacqueline Andréia Bernardes Leão-Cordeiro, Katarinne Lima Moraes, Karina Suzuki, Virginia Visconde Brasil, Antonio Márcio Teodoro Cordeiro Silva

**Affiliations:** 1 Universidade Federal de Goiás, Faculdade de Enfermagem, Goiânia, GO, Brasil.; 2 Bolsista da Coordenação de Aperfeiçoamento de Pessoal de Nível Superior (CAPES), Brasil.; 3 Universidade de Brasília, Faculdade de Ceilândia, Brasília, DF, Brasil.; 4 Pontifícia Universidade Católica de Goiás, Escola de Ciências Médicas, Farmacêuticas e Biomédicas, Goiânia, GO, Brasil.

**Keywords:** Cardiopulmonary Resuscitation, Heart Arrest, Out-of-Hospital Cardiac Arrest, Information Literacy, Health Literacy, Prehospital Care, Reanimação Cardiopulmonar, Parada Cardiorrespiratória, Parada Cardiorrespiratória Extra-Hospitalar, Competência em Informação, Letramento em Saúde, Assistência Pré-Hospitalar, Reanimación Cardiopulmonar, Paro Cardíaco, Paro Cardíaco Extrahospitalario, Alfabetización Informacional, Alfabetización en Salud, Atención Prehospitalaria

## Abstract

**Objective:**

to analyze the quality indicators and technical content of the videos for lay people posted on the YouTube platform, on cardiopulmonary resuscitation in adults and their audiovisual production regarding the principles of digital health literacy.

**Method:**

a descriptive and exploratory study, which selected videos recorded between December 2015 and April 2021. They were analyzed by indicators of the production of audiovisual material, considering the American Heart Association guidelines and the principles of digital health literacy. Descriptive and inferential statistics were performed.

**Results:**

of the 121 videos analyzed, 26 did not comply with any indicator on cardiopulmonary resuscitation, four reached 81% compliance, eight videos reached 79%, nine reached 69% and 74 videos, from 6% to 63%. According to the principles of digital health literacy, one video met 85% of the indicators, 81 met from 50% to 80% and 39, from 10% to 49%. A positive correlation was identified between literacy and cardiopulmonary resuscitation.

**Conclusion:**

no video presented 100% compliance with the American Heart Association guidelines. The absence of mechanisms for supervision and control over health-related contents allows for the posting of mistaken videos, which have been used as a learning method by people and can thus miss their greatest goal: save lives.

## Resumo

**Objetivo:**

analisar indicadores de qualidade e conteúdo técnico dos vídeos postados na plataforma *YouTube*, para leigos, sobre reanimação cardiopulmonar em adultos e sua produção audiovisual quanto aos princípios do letramento digital em saúde.

**Método:**

estudo descritivo, exploratório, que selecionou vídeos gravados entre dezembro de 2015 e abril de 2021. Foram analisados por indicadores da produção de material audiovisual, considerando as diretrizes da *American Heart Association* e os princípios do letramento digital em saúde. Foi realizada estatística descritiva e inferencial.

**Resultados:**

dos 121 vídeos analisados, 26 não atenderam qualquer indicador sobre ressuscitação cardiopulmonar; quatro atingiram 81% de conformidade; oito vídeos, 79%; nove vídeos, 69% e 74 vídeos de seis a 63%. De acordo com os princípios do letramento digital em saúde, um vídeo atendeu 85% dos indicadores; 81 vídeos atenderam de 50 a 80% e 39 vídeos, de 10 a 49%. Foi identificada correlação positiva entre letramento e ressuscitação cardiopulmonar.

**Conclusão:**

nenhum vídeo apresentou 100% de conformidade com as diretrizes da *American Heart Association.* A falta de mecanismos de fiscalização e controle sobre conteúdos relacionados à saúde permite a publicação de vídeos equivocados, que têm sido utilizados como aprendizado pelas pessoas e podem perder o maior objetivo que é salvar vidas.

## Resumen

**Objetivo:**

analizar los indicadores de calidad y contenido técnico de los videos publicados en la plataforma *YouTube*, para legos, sobre reanimación cardiopulmonar en adultos y su producción audiovisual según los principios de la alfabetización digital en salud.

**Método:**

estudio descriptivo, exploratorio, que seleccionó videos grabados entre diciembre de 2015 y abril de 2021. Fueron analizados en función de los indicadores de producción de material audiovisual, considerando las directrices de la *American Heart Association* y los principios de la alfabetización digital en salud. Se realizó estadística descriptiva e inferencial.

**Resultados:**

de los 121 videos analizados, 26 no cumplieron con ninguno de los indicadores de reanimación cardiopulmonar; cuatro lograron un 81% de conformidad; ocho videos, 79%; nueve videos, 69% y 74 videos de seis a 63%. De acuerdo con los principios de la alfabetización digital en salud, un video cumplió con el 85% de los indicadores; 81 videos cumplieron del 50 al 80% y 39 videos del 10 al 49%. Se identificó una correlación positiva entre la alfabetización y la reanimación cardiopulmonar.

**Conclusión:**

ningún video cumplió el 100% de las directrices de la *American Heart Association*. La falta de mecanismos de supervisión y control sobre los contenidos relacionados con la salud permite la publicación de videos erróneos, que han sido utilizados como experiencia de aprendizaje por las personas y es probable que no cumplan con el principal objetivo, que es salvar vidas.

**Highlights:**

(1) Non-compliance with the American Heart Association guidelines in the videos analyzed.

(2) Weaknesses and misconceptions in the diverse information on Cardiopulmonary Resuscitation.

(3) Lack of supervision and control over the health-related posts.

(4) Lack of connection between the ethical and technical attitude to ensure adequacy of the material.

(5) Non-identification of authorship in most of the videos.

## Introduction

Cardiopulmonary Arrest (CPA) consists in the interruption of blood and respiratory supply due to lack of heartbeats or to their ineffectiveness. It occurs suddenly and, in most cases, in non-hospital environments. If there is no fast and effective care, it may cause death or leave serious sequelae that will compromise the person’s quality of life^1^. This condition can be reversible if cardiopulmonary resuscitation (CPR) is performed, which consists in an organized sequence of maneuvers that will restore spontaneous bloodstream, properly and at the right moment^2^.

These maneuvers should be initiated by those who witness the CPA or first approach the victim. Usually, lay people have this initial contact and need to know how to act quickly, in order to contribute to survival and reduce the risk of injury to the victims^3^ ^-^ ^4^.

The Internet has been an immediate source of health information, which often does not consider people’s digital health literacy (DHL)^5^. It is defined as “the ability to seek, find, understand and evaluate diverse health information from electronic sources and apply the knowledge acquired to address or solve a health problem”^6^. In addition to that, non-screening of the quality of the materials posted results in a high risk of misinformation^7^.

One of the most popular and accessed online platforms with billions of views every day is YouTube, emerging as a significant and vast source of health information^8^ ^-^ ^9^. Considering the number of people who access educational videos for health and the absence of quality criteria for posts, concern arose to evaluate the diverse information related to CPR, available in this virtual environment.

In addition to considering the individual skills on access and use of the diverse information made available digitally, the material presented must contain information based on updated scientific evidence, be easily accessible and understandable^10^. The concern in relation to contents based on protocols and scientific guidelines is grounded on the possibility of misinformation about what is being transmitted to the people who use the Internet as a learning means.

Thus, this study aimed at analyzing the quality indicators and technical content of videos on cardiopulmonary resuscitation in adults and their audiovisual production related to the principles of digital health literacy, posted on the YouTube platform and aimed at a lay target audience.

## Method

### Design, locus and data collection period

This is an exploratory and descriptive research study with a quantitative approach. Data collection was carried out on the YouTube video content sharing platform (http://www.youtube.com.br). This platform was chosen among other websites because it is the most used research social media in Brazil and the second most visited site in the world^11^.

Data collection was carried out between 2020 and 2021, including videos recorded between December 2015 and April 2021. The content of the CPR videos was evaluated based on the American Heart Association guidelines^12^ ^-^ ^13^_,_ which are international references systematically updated every five years. Periodic updating of the AHA guidelines during data collection did not interfere with evaluation of the videos, as they were directed to the health professionals’ performance.

Videos on cardiopulmonary resuscitation in adults published in Portuguese and lasting a maximum of 4 minutes were included. “Short” videos are the most accessed by YouTube users^14^ ^-^ ^15^, with greater potential to be viewed and thus contributing to the educational aspects related to cardiopulmonary resuscitation^16^. The short length of videos and images evidences a change in the visualization and thinking patterns, making the products desirable for those who seek this information^17^. Videos that did not directly refer to CPR for lay people were excluded.

### Data collection and analysis

As a first step, a search was carried out in the Health Sciences Descriptors to identify the controlled descriptors related to the topic, namely: “cardiopulmonary reanimation”, “cardiopulmonary resuscitation” and “cardiopulmonary resuscitation for lay people”.

Subsequently, filters offered by the website itself were applied that delimited the search results: “type of result”, “videos” and “length” (selecting those of “short length - less than 4 minutes”).

A total of 350 videos on cardiopulmonary resuscitation were identified, with exclusion of 160 videos on CPR in infants, 52 in children and 17 videos that addressed topics other than CPR.

The videos selected for analysis were watched individually and recorded in a Microsoft Excel^®^ spreadsheet for data tabulation. The spreadsheet contained diverse information such as: length of the video; the post’s date; total views; number of likes and dislikes; authorship reliability; and the videos’ URL (Uniform Resource Locator) addresses, in order to assemble the display list and enable as many visits as necessary.

To guide data collection, the authors prepared two checklists, one to evaluate pertinence of the content considering the American Heart Association current guidelines^12^ ^-^ ^13^, and another to verify adequacy of the videos considering the criteria established for the production of educational materials in digital media^11^ ^,^ ^17^.

In order to ensure greater reliability of the data collected, the checklists were submitted to an evaluation by professionals working in the areas of cardiopulmonary resuscitation and health literacy.

The checklist on CPR was evaluated by 10 professionals, nurses and physicians with at least two years of experience in the areas of urgency, emergency or intensive care. The checklist on digital health literacy was evaluated by 10 researchers with training in Nursing, Medicine and Nutrition, who develop research focused on this theme. 

The evaluators analyzed the checklists stating whether or not they agreed with the items presented and if they had suggestions for changes, additions or exclusion of any item. They agreed with the items listed and suggested adding 02 items to the CPR checklist and 03 items to the DHL checklist. The final version of the checklist on CPR had 16 analysis items and the one for DHL had 20 items. Both have the following answer options: “0” for not meeting the criteria evaluated and “1” for the evaluation criteria met.

To analyze adequacy of the videos to the principles of digital health literacy, the following criteria were considered: a) title/description of the video; b) reliability of the video (presence of a health professional or unknown author or video produced by a company); c) proportion of likes/dislikes, in order to analyze the repercussion of the content presented; d) quality of the file (technical quality of the audio and image); e) verbal teaching quality (narrator/instructor is clear in the explanations); and f) visual teaching quality (complementary visual material, such as animations, subtitles or graphic elements)^18^.

### Data analysis

All variables of the video evaluation instrument were stored in an electronic spreadsheet, with the aid of Microsoft Excel^®^. Descriptive statistics were performed. For the categorical variables (number of items in the instruments), the absolute and relative percentage frequencies were determined. For the continuous variables (performance of the videos: likes and dislikes), central tendency and dispersion measures were calculated with the aid of the BioEstat^®^ software, version 5.3. In addition to that, inferential statistics were performed with application of Pearson’s correlation test, adopting a 5% significance level.

## Results

**Figure 1:**
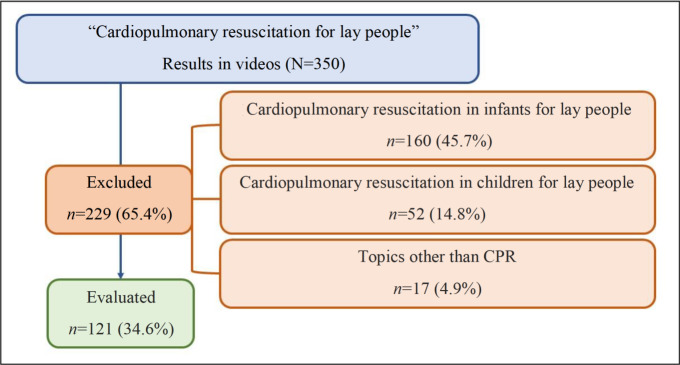
Flowchart corresponding to the selection process of videos on cardiopulmonary resuscitation in adults on the YouTube platform, intended for lay people

Of the 350 videos identified on the YouTube platform, 34.6% met the eligibility criteria. The flowchart in Figure 1 shows the stages of the data collection process, which consisted in the identification, screening and selection of the videos evaluated.

Of the 121 videos analyzed, 40.5% were produced by health professionals and students. The frequency of items met in relation to the evaluation of the diverse information on CPR is presented in Table 1.

**Table 1:** Frequency of items met in the evaluation of the content on cardiopulmonary resuscitation in adults, in 121 videos available on the YouTube platform, aimed at the lay population

No.	Items (N=121)	Yes		No	
		*n*	*f(%)*	*n*	*f(%)*
01	It provided guidance on site safety verification	22	18.2	99	81.8
02	It checked responsiveness	76	62.8	45	37.2
03	It checked breathing/chest expansion verification	63	52.1	58	47.9
04	It provided guidance on the emergency service request	71	58.7	50	41.3
05	It advised to start chest compressions, in case of absent breathing	73	60.3	48	39.7
06	It provided guidance on the need for the maneuver to be performed on a flat/rigid surface	22	18.2	99	81.8
07	It followed the C-A-B sequence	43	35.5	78	64.5
08	It provided guidance on the proper location of the hands in chest compressions	59	48.8	62	51.2
09	It dealt with correct positioning of the hands	44	36.4	77	63.6
10	It provided guidance on a minimum depth of the compressions of 2 inches (5 cm)	40	33.1	81	66.9
11	It reinforced the importance of chest return	16	13.2	105	86.8
12	It provided guidance on the request for the Automatic External Defibrillator (AED)	21	17.4	100	82.6
13	It addressed speed of the compressions (from 100/min to 120/min)	51	42.1	70	57.9
14	It provided guidance on minimizing interruptions in compressions	09	7.4	112	92.6
15	It provided guidance on continuing compressions until arrival of rescue team	40	33.1	81	66.9
16	It provided guidance on when to stop the compressions	31	25.6	90	74.4

The items most frequently met in the videos were as follows: checking responsiveness (62.8%); chest compression initiation if absent breathing (60.3%); guidance on emergency service request (58.7%) and checking breathing/chest expansion verification (52.1%). The least met items were: minimization of interruptions in compressions (92.6%); reinforcement of the importance of chest return (86.8%); request for the Automatic External Defibrillator (AED) (82.6%); guidance on the site safety verification (81.8%); maneuvers performed on a flat/rigid surface (81.8%); and when to stop the chest compressions (74.4%).

**Figure 2:**
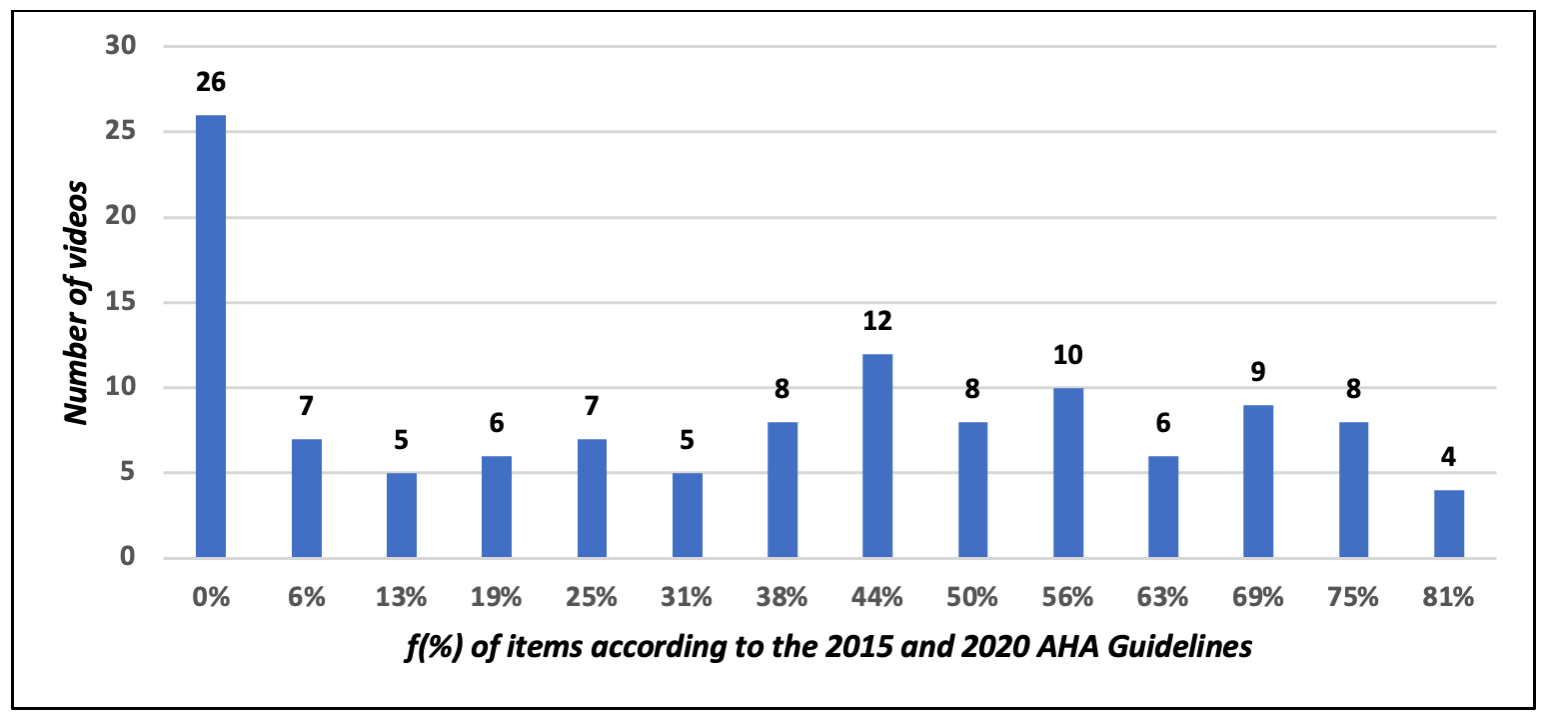
Frequency of videos and compliance of the content related to the American Heart Association guidelines, available on YouTube

No video had 100% compliance with the American Heart Association guidelines^12^ ^-^ ^13^. Nearly 26 videos did not meet the items in the instrument on CPR, four reached 81% compliance, eight reached 79%, nine met 69% of the items and 74 videos reached from 6% to 63% compliance. The number of videos in accordance with the AHA guidelines can be identified in Figure 2.

Of the 121 videos analyzed, the items that obtained the highest compliance frequency were the following: the production date of the video is included in the video description field (95.9%); sound and image coincided (95.0%); lighting was adequate (90.1%); and the author used means to contribute to didactics (88.4%).

The least met items were the following: providing information sources related to the content in the video description field (6.6%); citing the main references used to produce the content disclosed in the video (7.4%); generating reflective and inferential questions to the viewers about the subject matter addressed (25.6%); and meeting the talking head with complementary material (28.1%) (Table 2).

**Table 2:** Frequency with which the items on digital health literacy were met in 121 videos available on the YouTube platform, aimed at the lay population

No.	Compliance with the items in the instrument (N=121)	Yes		No	
		*n*	*f(%)*	*n*	*f(%)*
1	The production date of the video is included in the video description field	116	95.9	5	4.1
2	The title of the video is informative, it clearly presents what will be addressed	87	71.9	34	28.1
3	The author of the video identifies him/herself (professional/student)	49	40.5	72	59.5
4	The author of the video identifies him/herself (name)	61	50.4	60	49.6
5	The author cites the main references used to produce the content disclosed in video	9	7.4	112	92.6
6	The author uses means to contribute to didactics (realistic simulation, blackboard, mannequin or slide)	107	88.4	14	11.6
7	The author of the video generates reflective and inferential questions to the viewers about the subject matter addressed	31	25.6	90	74.4
8	It provides/makes available sources of diverse content-related information in the video description field	8	6.6	113	93.4
9	Sound quality is adequate, what is spoken is heard and understood	96	79.3	25	20.7
10	It is a talking head with complementary material (video in which the person speaks to the camera, but is interrupted by other images, animations, graphs, illustrations)	34	28.1	87	71.9
11	Sound and image coincide	115	95.0	6	5.0
12	It has subtitles	87	71.9	34	28.1
13	Clear and objective language	70	57.9	51	42.1
14	The images (figures, etc.) are clear and depict the content covered	106	87.6	15	12.4
15	Adequate lighting (it allows for good visualization of the content)	109	90.1	12	9.9
16	No noise or music interfering with understanding of the content during speech	105	86.8	16	13.2
17	The author of the video is linked to some educational or care institution related to the subject matter	74	61.2	47	38.8
18	The video presents the symbol of the institution to which the author is linked	50	41.3	71	58.7
19	If the video contains a written part, the font size used is easy to view	61	50.4	60	49.6
20	The diverse information presented in the video follows national or international content protocols	62	51.2	59	48.8

**Figure 3:**
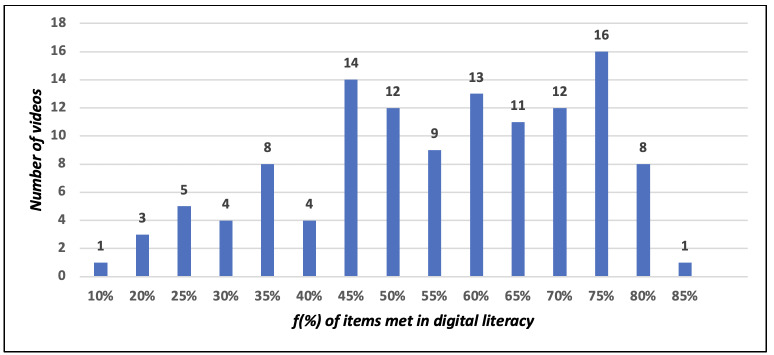
Frequency of the videos and compliance with the principles of digital health literacy, available on YouTube

It was observed that one video met 85% of checklist items, 81 videos met from 50% to 80%, and 39 met from 10% to 49% of the checklist. Figure 3 shows the number of videos according to the percentage of adequacy to the health literacy recommendations.

The literacy assessment items and the CPR content assessment items addressed in the videos were added up separately and submitted to Pearson’s correlation test. A positive correlation (*p*<0.0001; r=0.3497) was observed between the literacy quality indicators and the CPR content. This means that the videos that met the audiovisual production indicators were those that presented the most appropriate content on CPR for lay people.

In relation to the videos’ performance on YouTube, the mean number of likes was 118.4, with a coefficient of variation (CV) of 441.2%, and variation from zero to 5,100 likes. The mean number of dislikes recorded was 2.5 (CV=334.8%), varying from zero to 56 dislikes. The mean number of views was 4,487 (CV=393.2%), varying from 4 to 147,886. Finally, the mean length of the videos was 2 minutes and 30 seconds (CV=46.5%).

## Discussion

It is a fact that videos are excellent didactic resources in the teaching-learning process and facilitate the dynamic propagation of diverse information. However, those who are willing to produce them should be aware of the quality of the content to be transmitted. In this context, analyzing the quality of the videos on cardiopulmonary resuscitation in one of the largest online platforms in the world made it possible to verify that the benefits of this social media can be reduced by the low level of digital health literacy of its users and by the neglect regarding the content posted^19^.

The results of the current study evidenced lack of mastery or attention on the correct way to act in a situation of Cardiopulmonary Arrest (CPA) by those who produced the videos, even when, in several cases, the authors are health professionals and students. Considering that most of the videos do not comply with the AHA guidelines^12^ ^-^ ^13^, it is also important to consider the ethical commitment to reliability of the information and to the paradigms pertinent to the standards, rules and guidelines^20^. 

Any useful, technically appropriate and ethical evaluation should guide the action strategy, considering that ethics permeates technique and social responsibility^21^. A number of Brazilian studies^22^ ^-^ ^24^ evaluated the quality of the diverse information on CPR posted on the YouTube platform, and verified that most of the videos did not include the guidelines in force at the time, corroborating the results of this research. 

Thus, those in charge of the dissemination of contents on virtual platforms, in contradiction with the recommendations made by the responsible agencies, urgently need to modify their behavior so that misleading or incomplete information is not disseminated, at the risk of causing significant harms to the lives of significant numbers of their users who resort to them as a learning source^8^ ^,^ ^25^.

Each stage of the cardiopulmonary resuscitation maneuver to be performed is fundamental. It is based on studies that justify its execution and function, with the main objective of manually maintaining the heart’s contractile function, ensuring blood perfusion to keep the organs oxygenated, for as long as possible until arrival of the rescue team, thus reducing the neurological and cardiac sequelae that may occur^26^.

Correct application of the CPR procedures was not observed in most of the videos, which generates concern in times when technology is gradually gaining more space each day and imposes changes in the learning process. People who watch these videos will perform the maneuver inappropriately, not achieving the greatest goal of saving lives^27^.

Nearly 60.3% of the videos provided guidance on performance of the compressions when noticing absence of breathing and chest expansion, but neglected how to perform them. They should be performed on a flat and rigid surface^28^, and the lower half of the sternum is the chest site for compression; the hands should be positioned one interlaced on the other; and the correct depth is 2 inches or 5 cm for adults, avoiding excess in the depth of chest compressions and fractures^12^.

In addition to that, the importance of chest return after the compressions in order not to compromise venous return and cardiopulmonary blood flow, adequate speed with a frequency of 100 to 120 compressions per minute in adult victims, non-interruption of CPR until arrival of the rescue team that will take the automatic external defibrillator, and site safety verification to avoid complications both in the victim and in the person who is providing assistance, represent essential information for adequate care in the face of a CPA situation^12^.

Absence of this information can lead the lay rescuers to errors and even interfere with the victims’ survival rate. It is known that the survival rate after a CPA event can triple when high-quality and early CPR is performed^29^.

Once again, this reinforces that most of the videos directed to lay people that are posted on YouTube on this theme should not be recommended as a learning method, although it is undeniable that YouTube videos can contribute to the teaching-learning process of the population. Thoughtful planning should be carried out, with control of the content conveyed by the digital platform, allowing it to be used with all the capacity it offers^16^ ^,^ ^30^.

One aspect to be considered in the elaboration of health-related videos, which may assist in the quality of the content to be posted, is to use the recommendations set forth in the guidelines and protocols in force with their respective care algorithms, generating videos with evidence-based information and the correct care sequence in CPR. These strategies are considered effective for safe care; and YouTube, which is considered the most widespread website among Internet users and used as a research source, should pay attention to this care in its posts^16^ ^,^ ^30^.

Currently, the Internet and social networks represent the main channels for seeking health information and peer support. However, the benefits of social media on health can be reduced by the low level of digital health literacy, and it is indispensable that the information is organized and accessible enough so that the users can find it and apply it for their own health^31^.

The videos that had the highest number of items met were the most viewed and with the highest number of likes. This indicates more acceptability by the users regarding the content. Credibility of the information is an indispensable attribute to ensure that the CPR techniques are especially adopted for the lay person, and that it requires basic digital literacy conditions.

In addition to lack of mastery of the technique, digital skills are also missing on the part of the authors, and the schools should start including the health literacy curriculum in their pedagogical plans in order to train professionals who are responsive to literacy. In addition to that, digital health is already a global reality and it is up to the training schools to prepare future professionals to meet the demands of this new reality, based on ethics and on the promotion of safe health in the digital environment^32^.

Both quality of the content and of the video production should be factors taken into consideration. Although it is not difficult to produce a video, the technical aspects must be taken into account, as the producers may not only designate materials to achieve particular purposes, but take responsibility for conveying information on the effective use of such materials, which may exert an impact on people’s lives^33^ ^-^ ^34^.

Most of the authors of the videos analyzed in this study did not inform if they were health professionals or students; many of them only reported their names. However, use of technical terms was observed; some were dressed as health professionals and used mannequins in the simulations; others were in educational institution environments, therefore presuming that they were health professionals or students. 

When posting content on a public access virtual platform, identification (name and profession) of those who are in charge of that post should be included in the material, as this information generates trust in the production^35^. 

A study^18^ pointed out that criteria that convey reliability to the videos and that guarantee maintenance of their quality are the identification and the production date of the video, as they inform the timeliness of the content presented. This same study pointed out that the objective of the video should be obvious to the viewer; it should have a title that clearly reflects its purpose; and in no case the title should be misleading about the content that will be presented^18^.

In situations where health terminologies are used, it is important that they are accessible to the audience to be reached; the necessary technical terms must be defined. In addition to that, it is important to reference the diverse information that is being used since, in addition to proving from where the information was extracted, it demonstrates scientific and ethical care. Appropriating content without giving due credit to the source is unethical and may result in legal actions^36^ ^-^ ^37^.

This study contributes important advances in scientific knowledge, as we are living in a new learning and knowledge acquisition scenario through digital platforms, especially during the pandemic period, when it is necessary to identify which health information is reliable or not. In this sense, health professionals can devise strategies such as a list of websites with reliable educational materials available to the population.

In addition to that, efforts should be made to promote health education programs that aim at improving the DHL conditions, enabling people to acquire the ability to access, understand and evaluate the diverse information available in the digital media.

This research presents limitations, such as the absence of validated instruments to evaluate educational videos containing the principles of digital health literacy. This gap can be explained by the fact that this is still a recent topic in Brazil, pointing out the need for further research studies. In addition, the possibility of analyzing longer videos on the subject matter since, even if less frequently, they are also viewed by lay people. 

This research showed that there is weakness in the health information available on cardiopulmonary resuscitation, as it is a topic that must be widely disseminated to the lay population, so that it can provide immediate help with correct maneuvers and supported by protocols and guidelines for the safety of all.

Thus, it is recommended that future studies develop and validate specific instruments with content and quality indicators of the videos, thus ensuring quality of the diverse information on websites, making assertive and evidence-based health decisions, with quality screening of information posted^38^ ^-^ ^39^. In addition to that, the number of videos related to CPR in children (60% of those found) points to prospects for future studies that analyze the quality of videos in this age group.

## Conclusion

The results evidenced weaknesses in the diverse information on cardiopulmonary resuscitation available on the YouTube platform, related to its reliability and quality. Much of the information that would be indispensable to ensure content credibility, defined by digital health literacy, was omitted: authorship, institutions to which the authors are linked and references.

Dynamism of the digital environment favors knowledge dissemination and is considered a great advance for the democratization of health information. However, the videos need to be based on scientifically defined protocols, so that users of the digital platform receive correct information and can achieve the greatest goal: save lives.

## References

[1] Marques SC, Dias DF, Aragão IPB (2019). Prevalence of knowledge and application of cardiopulmonary resuscitation techniques. Rev Fluminense Extensão Universitária.

[2] Abelsson A, Nygardh A (2019). To enhance the quality of CPR performed by youth layman. Int J Emerg Med.

[3] Maciel AO, Roseno BR, Cavalcanti EO, Rodrigues NS, Santos LC (2020). Knowledge assessment regarding cardiorespiratory arrest and choking among teachers and students at a public school in the Federal District. Braz J Develop.

[4] Terassi M, Borges AKPG, Garanhani ML, Martins EAP (2015). The perception of children of elementary education about cardiorespiratory arrest. Semina Cienc Biol Saude.

[5] Meppelink CS, van Weert JCM, Haven CJ, Smit EG (2015). The effectiveness of health animations in audiences with different health literacy levels: an experimental study. J Med Internet Res.

[6] Norman CD, Skinner HA (2006). eHEALS: The eHealth Literacy Scale. J Med Internet Res.

[7] Manning DL, Dickens C (2006). Health literacy: more choice, but do cancer patients have the skills to decide?. Eur J Cancer Care (Engl).

[8] Katipoglu B, Akbas I, Koçak AO, Erbay MF, Turan EI, Kasali K (2019). Assessment of the accuracy of cardiopulmonary resuscitation videos in English on YouTube according to the 2015 AHA Resuscitation Guidelines. Emerg Med Int.

[9] Chintalapati N, Daruri VKS (2017). Examining the use of YouTube as a learning resource in higher education: scale development and validation of tam model. Telemat Inform.

[10] Turkdogan S, Schnitman G, Wang T, Gotlieb R, How J, Gotlieb WH (2021). Development of a digital patient education tool for patients with cancer during the COVID-19 pandemic. JMIR Cancer.

[11] Langford A, Loeb S (2019). Perceived patient-provider communication quality and sociodemographic factors associated with watching health-related vídeos on YouTube: a cross-sectional analysis. J Med Internet Res.

[12] American Heart Association (2015). Destaques da American Heart Association 2015: atualização das diretrizes de RCP e ACE.

[13] American Heart Association (2020). Destaques das diretrizes de RCP e ACE de 2020 da American Heart Association.

[14] Schneider CK, Caetano L, Ribeiro LOM (2021). Analysis of educational videos on YouTube characters and legibility. Rev Renote.

[15] Pellegrini DP, Reis DD, Monção PC, Oliveira R YouTube: uma nova fonte de discursos.

[16] Salvador PTCO, Costa TD, Gomes ATL, Assis YMS, Santos VEP (2017). Patient safety: characterization of YouTube videos. Rev Gaúcha Enferm.

[17] Vasconcellos RR (2019). As mídias sociais audiovisuais breves: estratégias e conteúdos de vídeos de até quinze segundos.

[18] Feller R (2018). Guidelines for the preparation and evaluation of video career media.

[19] Atique S, Hosueh M, Fernandez-Luque L, Gabarron E, Wan M, Singh O (2016). Lessons learnt from a MOOC about social media for digital health literacy. Annu Int Conf IEEE Eng Med Biol Soc.

[20] Dawkins-Moultin L, McKyer L, McDonald A (2019). Health literacy competence of health education students in three universities. Pedagogy Health Promot.

[21] Minayo MCS (2005). Avaliação por Triangulação de métodos: abordagem de programas sociais.

[22] Tourinho FSV, Medeiros KS, Salvador PTCO, Castro GLT, Santos VEP (2012). Analysis of the YouTube videos on basic life support and cardiopulmonary resuscitation. Rev Colégio Bra Cirurgiões.

[23] Costa FRR, Moreira DMT, Carneiro SDRM, Viana FAC, Lima DLF, Santos SE (2015). Evaluation of Basic Life Support (BLS) videos published on YouTube. Rev Cir Traumatol Buco-Maxilo-Fac.

[24] Pinto APCM, Dantas MSP, Salvador PTCO, Martins CCF, Santos VEP (2015). Analysis of YouTube videos addressing the indwelling urinary catheterization procedure in women. Rev Cogitare Enferm.

[25] Salvador PTCO, Costa TD, Gomes ATL, Assis YMS, Santos VEP (2017). Patient safety: characterization of YouTube videos. Rev Gaúcha Enferm.

[26] Monsieurs KG, Nolan JP, Bossaert LL, Grief R, Maconochie IK, Nikolaou NI (2015). European Resuscitation Council Guidelines for Resuscitation 2015. Resuscitation.

[27] Vidal AS, Miguel JR (2020). Digital technologies in contemporary education. Rev Multidisciplinar Psicol.

[28] Vianna CA, Oliveira HC, Souza LC, Silva RC, Brandão MAG, Campos JF (2021). Impact of compression surfaces on cardiac massage during cardiopulmonary reanimation: an integrative review. Esc Anna Nery.

[29] Falcão LFR, Ferez D, Amaral JLG (2011). Atualização das diretrizes de ressuscitação cardiopulmonar de interesse ao anestesiologista. Rev Bras Anestesiol.

[30] Tulgar S, Selvi O, Serifsoy TE, Senturk O, Ozer Z (2017). YouTube as an information source of spinal anesthesia, epidural anesthesia and combined spinal and epidural anesthesia. Rev Bras Anestesiol.

[31] Boeres S (2018). Literacy and digital information attached to lifelong learning. Rev Digital Biblioteconomia Ciên Inform.

[32] Saunders C, Palesy D, Lewis J (2019). Systematic Review and Conceptual Framework for Health Literacy Training in Health Professions Education. Health Prof Educ.

[33] Fernandes ICF, Siqueira KM, Barbosa MA (2018). Assessment of videos about the inhalation technique for childhood asthma: educational or mediatic?. Rev Eletr Enferm.

[34] Villa LSC, Mello ADC, Gonçalves JV, Silva TMG, Bernuci MP (2021). Quality assessment of the most watched breast cancer videos on YouTube: relevance to improve women's health. Rev Eletr Comun Inform Inov Saúde.

[35] Van Den Beemt A, Thurlings M, Willems M (2020). Towards an understanding of social media use in the classroom: a literature review. Technol Pedag Educ.

[36] Pithan LH, Vidal TRA (2013). Academic plagiarism as an ethical, legal and teaching problem. Direito Justiça.

[37] Brixey JJ, Newbold SK (2017). Nursing informatics pioneers embrace social media. Stud Health Technol Inform.

[38] França T, Rabello ET, Magnago C (2019). Digital media and platforms in the Permanent Health Education field: debates and proposals. Rev Saúde Debate.

[39] REDE Interagencial de Informação para a Saúde (2008). Indicadores básicos para a saúde no Brasil: conceitos e aplicações.

